# Factors associated with impaired quality of life three months after being diagnosed with COVID-19

**DOI:** 10.1007/s11136-021-02998-9

**Published:** 2021-09-28

**Authors:** Verena Rass, Bogdan-Andrei Ianosi, Laura Zamarian, Ronny Beer, Sabina Sahanic, Anna Lindner, Mario Kofler, Alois Josef Schiefecker, Philipp Mahlknecht, Beatrice Heim, Victoria Limmert, Thomas Sonnweber, Alex Pizzini, Piotr Tymoszuk, Christoph Scherfler, Atbin Djamshidian, Stefan Kiechl, Ivan Tancevski, Klaus Seppi, Bettina Pfausler, Judith Loeffler-Ragg, Raimund Helbok

**Affiliations:** 1 Department of Neurology, Medical University of Innsbruck, Anichstrasse 35, 6020 Innsbruck, Austria; 2 Department of Internal Medicine II, Medical University of Innsbruck, Anichstrasse 35, 6020 Innsbruck, Austria

**Keywords:** COVID-19, SARS-CoV-2, Quality of life, Neuro-COVID, Mental health

## Abstract

**Purpose:**

To assess patient characteristics associated with health-related quality of life (HR-QoL) and its mental and physical subcategories 3 months after diagnosis with COVID-19.

**Methods:**

In this prospective multicentre cohort study, HR-QoL was assessed in 90 patients using the SF-36 questionnaire (36-item Short Form Health Survey), which consists of 8 health domains that can be divided into a mental and physical health component. Mental health symptoms including anxiety, depression, and post-traumatic stress disorders were evaluated using the Hospital Anxiety and Depression Scale (HADS) and Post-traumatic Stress Disorder Checklist-5 (PCL-5) 3 months after COVID-19. Using descriptive statistics and multivariable regression analysis, we identified factors associated with impaired HR-QoL 3 months after COVID-19 diagnosis.

**Results:**

Patients were 55 years of age (IQR, 49–63; 39% women) and were classified as severe (23%), moderate (57%), or mild (20%) according to acute disease severity. HR-QoL was impaired in 28/90 patients (31%). Younger age [per year, adjOR (95%CI) 0.94 (0.88–1.00), *p* = 0.049], longer hospitalization [per day, adjOR (95%CI) 1.07 (1.01–1.13), *p* = 0.015], impaired sleep [adjOR (95%CI) 5.54 (1.2–25.61), *p* = 0.028], and anxiety [adjOR (95%CI) 15.67 (3.03–80.99), *p* = 0.001) were independently associated with impaired HR-QoL. Twenty-nine percent (*n* = 26) scored below the normal range on the mental health component of the SF-36 and independent associations emerged for anxiety, depression, and self-reported numbness. Impairments in the physical health component of the SF-36 were reported by 12 (13%) patients and linked to hypogeusia and fatigue.

**Conclusion:**

Every third patient reported a reduction in HR-QoL 3 months after COVID-19 diagnosis and impairments were more prominent in mental than physical well-being.

**Supplementary Information:**

The online version contains supplementary material available at 10.1007/s11136-021-02998-9.

## Introduction

The Coronavirus disease 2019 (COVID-19) manifests in a high variety of symptoms, involving almost all organs ranging from asymptomatic to critically ill patients with fatal outcomes [1]. A range of neurological and psychiatric sequalae such as headache, hyposmia, myalgia, neuropathy, encephalopathies, neurocognitive impairment, anxiety, and depressed mood in patients who have recovered from COVID-19 have been reported [2]. Underlying pathophysiologic mechanisms for these manifestations include virus-associated effects (e.g. direct invasion to the central nervous system, endotheliopathy, vasculitis, prothrombotic effects, or indirect virus-associated mechanisms, such as inflammation and immune-mediated toxicity), and disease-associated complications, especially for severe patients with prolonged ICU stay [2, 3]. When investigating health-related quality of life (HR-QoL) and mental health symptoms, impairments such as anxiety, depression, and post-traumatic stress disorders, as well as sleep disorders and persistent fatigue, were identified in a substantial proportion of patients even months after COVID-19 [4–12]. In this regard, a decline in HR-QoL compared to baseline was reported after 3 and 6 months in a considerable number of patients ranging from 33 to 72% at 3 months [13, 14], and 31% to 54% at 6 months [12, 15]. Similarly, HR-QoL was diminished during other disease outbreaks such as H1N1 [16, 17] or epidemic Influenza A (H7N9) [18] compared to the general population with the potential to recover after 3 to 6 months [16, 17]. Risk factors for impaired HR-QoL included hospitalization, lower social class, any comorbidity, and Acute Respiratory Distress Syndrome (ARDS) during the acute disease [16, 17].

The SF-36 is an established tool to measure HR-QoL, and it represents the patients’ perspectives on their health status and provides 8 health domains that can be divided into a physical component summary (PCS) and a mental component summary (MCS) [19].

The high number of COVID-19 cases worldwide with over 173 million confirmed cases by 08th June 2021 [20] makes studying impaired quality of life on the long-term health important.

This study, therefore, aims to investigate the association of cardio-pulmonary as well as neurological and psychiatric symptoms, evaluated at the 3-month follow-up, with impaired HR-QoL as assessed with the SF-36 3 months after COVID-19 diagnosis.

## Methods

### Study design, setting, and participants

The study design was guided by the STROBE statement on observational cohort studies. This is a prospective multicentre cohort study including consecutive COVID-19 patients managed at three Tyrolean medical centres (Austria) during the acute phase of the disease, namely at one tertiary (Innsbruck), one secondary (Zams) care centre, and one acute rehabilitation facility (Muenster) [21]. The diagnosis of COVID-19 conformed to WHO criteria and was based on a typical clinical presentation including dyspnoea, cough, fever, and a positive RT-PCR SARS-CoV-2 test from a nasopharyngeal or oropharyngeal swab. Overall inclusion criteria were (1) confirmed COVID-19, (2) age ≥ 18 years, and (3) hospitalization or outpatient management with persistent symptoms 6 weeks after the diagnosis. Out of 190 patients screened during the acute phase, 145 were included in the “Development of Interstitial Lung Disease in Patients with SARS-CoV-2 infection” (CovILD) study and 135 underwent in-person neurological follow-up 3 months after baseline, defined as the day of diagnosis by a positive SARS-CoV-2 test result between April and September 2020 [21, 22]. Ninety patients agreed to fill out questionnaires (HADS, SF-36) and were, therefore, included in the current sub-analysis.

The local ethics committee approved the conduct of the study (Medical University of Innsbruck, EK Nr: 1103/2020), which was registered at ClinicalTrials.gov (NCT04416100). All patients gave written informed consent according to local regulations and study procedures conformed to the 1964 Declaration of Helsinki.

### Study procedures and data collection

All patients received a cardio-pulmonary follow-up and were screened for neurological deficits in a structured in-person examination 3 months after laboratory confirmed diagnosis. Cardio-pulmonary symptoms were evaluated by internal medicine specialists and intensivists, while neurological evaluations were performed by neurological consultants or residents under supervision by neurologists. Patients were interviewed for pre-medical history as well as for current medication. Details were reported previously [21, 22].

For the objective assessment of olfactory function to diagnose hyposmia, we used the 16-item Sniffin' Sticks test (SS-16; Burghart Medizintechnik, Germany). Hyposmia was defined by the correct identification of ≤ 12 odours, as per manufacturer criteria. Cognitive impairment was classified as patients scoring < 26/30 points on the Montreal Cognitive Assessment (MoCA) [23]. Post-traumatic stress disorders (PTSD) were assessed with the Post-traumatic Stress Disorder Checklist-5 (PCL-5) [24] which captures 20 symptoms (each 0 to 4 points) and where scores > 32 are indicative of a clinically relevant PTSD. Anxiety and depression were evaluated with the Hospital Anxiety and a Depression Scale (HADS) questionnaire [25]. It consists of an anxiety (HADS-A) and depression (HADS-D) subscale, each containing 7 items that score from 0 to 3. Accordingly, scores range from 0 to 21 in each subscale. Scores > 7 suggest mild disorder and were used for the current analyses [26]. Fatigue was assessed by self-report. Internal reliability of self-report measures including self-reported fatigue, impaired concentration, sleep disturbance, new headache, and numbness was high (Cronbach’s Alpha = 0.622). Functional outcome 3 months after the acute disease was assessed with the Glasgow Outcome Scale Extended (GOSE) and the modified Rankin Scale Score (mRS) [21].

Concerning cardio-pulmonary health 3 months after COVID-19 diagnosis, examinations included the assessment of the Modified British Medical Research Council (mMRC) dyspnoea score, lung function testing encompassing spirometry, body plethysmography, diffusion capacity for carbon monoxide (DLCO) adjusted for haemoglobin, trans-thoracic echocardiography, standard laboratory examinations, and a low-dose computed tomography (CT) scan of the chest. Classification of CT abnormalities was based on pulmonary findings in each lobe ranging from 0 (none) to 5 (massive findings, parenchymal destructions) resulting in a maximum score of 25 [22].

Disease severity groups were defined according to the required invasiveness during the acute disease: (1) non-hospitalized (mild) patients, (2) hospitalized (moderate) patients not requiring ICU admission, and (3) severe COVID-19 patients admitted to the ICU.

### Outcome measures

Health-related quality of life assessed with the 36-item Short Form (SF-36v2) was the primary outcome measure of this study. The SF-36v2 is a self-report questionnaire and rates the subjective health condition [19, 27, 28]. It provides scores of 8 health domains: Physical functioning, physical role functioning, bodily pain, general health perceptions, vitality, social role functioning, emotional role functioning, and mental health. Each subscale ranges between 0 and 100 points. These domains can be classified into the physical component summary (PCS) and mental component summary (MCS) each ranging from 0 to 100 points. Higher levels indicate a better health condition. Scores below 40 are considered impaired according to norm-based scoring.

### Statistical analysis

Categorical variables are given as count and percentage, continuous variables as median, and interquartile range or mean and standard deviation. Based on variable distribution (tested by the Kolmogorov–Smirnov test and Shapiro–Wilk test), parametric and non-parametric procedures including the *t* test, Mann–Whitney *U* test, and Kruskal–Wallis test were used to assess differences between categories of disease severity (mild, moderate, and severe), and patients with or without mental health symptoms. Categorical variables were analysed using the Chi-squared and Fishers exact test.

The mental health burden was estimated by a point score (range 0–3) with one point each given for a HADS-A > 7, HADS-D > 7, and PCL-5 > 32 [29]. Similarly, the physical symptom burden was calculated by adding points for persistent dyspnoea, cough, objective hyposmia (SS-16 ≤ 12), new headache, vertigo, myalgia, polyneuromyopathy, impaired cognition (MoCA < 26), self-reported poor concentration, sleep disorder, and fatigue (one point each), resulting in a maximum of 11 points. To assess the association between the mental health burden/physical symptom burden and the mental as well as physical component summary of the SF-36, we calculated generalized linear models using a "quasipoisson" link function. The corresponding incidence rate ratios (IRR) and standard errors (SE) were calculated using the mfx (version 1.2–2) package.

To assess independent associated factors with poor HR-QoL, multivariable logistic regression analysis was employed, and for model selection, we used a multi-step approach. We selected clinical meaningful variables to build our full model, using a stepwise selection based on the Akaike information criterion (AIC). For this analysis, we used the R Package MASS (version 7.3–53.1) with the StepAIC function and calculated adjusted odds ratios with 95% confidence intervals. A two-sided *p* value < 0.05 was considered statistically significant. All analyses and graphical representations were performed with SPSS (IBM SPSS Statistics, Version 24.0 Armonk, NY, USA) and R version 4.0.2.

## Results

Ninety out of 135 (67%) patients responded to the SF-36 questionnaire 100 (IQR 90–109) days after disease diagnosis and were included in the current analysis. Patients were median 55 (IQR 49–63) years of age, 39% were women and represented all severity grades during the acute phase of infection (severe: 23%, moderate: 57%, mild: 20%). Detailed demographic information is specified in Table 1. Characteristics were not significantly different between included and excluded patients (*p* > 0.05).

**Table 1:** Demographics, comorbidities, and therapy in 90 COVID-19 patients according to COVID-19 severity

	*n* = 90	Severe disease requiring ICU admission *n* = 21 (23%)	Moderate severity (Hospitalization- non-ICU) *n* = 51 (57%)	Mild severity (Outpatient) *n* = 18 (20%)	*p* value*
Age (years)^a^	55 (49–63)	55 (54–62)	57 (51–69)	48 (39–54)	0.003
Sex (female)^c^	35 (39)	5 (24)	19 (37)	11 (61)	0.055
Ethnicity; Caucasian^c^	90 (100)	21 (100)	51 (100)	18 (100)	–
Body mass index (SI)^a^	26 (24–29)	25 (24–28)	27 (25–30)	25 (21–27)	0.046
Current smoking^c^	2 (2)	0 (0)	2 (4)	0 (0)	0.457
Ex-smoking^c^	54 (40)	5 (24)	25 (49)	4 (22)	0.042
Pack years^b^	7 ± 14	4 ± 7	11 ± 17	1 ± 3	0.009
**Premedical history**					
Cardiovascular disease^c^	32 (36)	12 (57)	20 (39)	0 (0)	0.001
Arterial hypertension^c^	23 (26)	10 (48)	13 (26)	0 (0)	0.003
Pulmonary disease^c^	17 (19)	4 (19)	11 (22)	2 (11)	0.622
Endocrinological disease^c^	41 (46)	12 (57)	27 (53)	2 (11)	0.004
Hypercholesterolemia^c^	17 (19)	4 (19)	13 (26)	0 (0)	0.060
Diabetes mellitus II^c^	14 (16)	4 (19)	10 (20)	0 (0)	0.126
Malignancy^c^	12 (13)	3 (14)	8 (16)	1 (6)	0.548
Immunological deficiency^c^	5 (6)	4 (19)	0 (0)	1 (6)	0.006
**Pre-existing neurological diseases**					
None^c^	69 (77)	17 (81)	38 (75)	14 (78)	0.835
Stroke^c^	0 (0)	0 (0)	0 (0)	0 (0)	–
Parkinsonism^c^	0 (0)	0 (0)	0 (0)	0 (0)	–
Multiple sclerosis^c^	0 (0)	0 (0)	0 (0)	0 (0)	–
Motor neuron disease^c^	0 (0)	0 (0)	0 (0)	0 (0)	–
(Poly)-Neuropathy^c^	5 (6)	1 (5)	4 (8)	0 (0)	0.451
Traumatic brain injury^c^	1 (1)	0 (0)	1 (2)	0 (0)	0.679
Restless legs syndrome^c^	2 (2)	1 (5)	0 (0)	1 (6)	0.259
Essential tremor^c^	2 (2)	1 (5)	1 (2)	0 (0)	0.592
Migraine^c^	3 (3)	1 (5)	1 (2)	1 (6)	0.702
Neuromuscular disease^c^	0 (0)	0 (0)	0 (0)	0 (0)	–
Epilepsy^c^	0 (0)	0 (0)	0 (0)	0 (0)	–
Other^c^	10 (11)	1 (5)	7 (14)	2 (11)	0.546
**Pre-existing psychiatric diseases**					
Depression (treated) ^c^	6 (7)	3 (14)	3 (6)	0 (0)	0.193
**Treatment and hospital course**					
Oxygen requirement^c^	49 (55)	21 (100)	28 (55)	0 (0)	< 0.001
Mechanical ventilation^c^	20 (22)	20 (95)	0 (0)	0 (0)	< 0.001
Steroid treatment^c^	15 (17)	7 (33)	8 (16)	0 (0)	0.023
Length of hospital stay (days)^a^	10 (5–19)	28 (19–38)	8 (5–11)	–	< 0.001
Early rehabilitation^c^	18 (20)	15 (71)	3 (6)	0 (0)	< 0.001
Length of rehabilitation (days)^a^	21 (21–27)	21 (21–27)	19–21	–	< 0.001

Based on data distribution data are given in ^a^median (interquartile range), ^b^mean ± standard deviation or ^c^counts (%)

^*^Chi-square or Kruskal–Wallis tests were used to assess for differences across severity grades (severe, moderate, mild). A P value < 0.05 signifies a significant different data distribution across severity groups

### Three-month prevalence of impaired HR-quality of life

HR-QoL as assessed with the SF-36 was impaired in 28/90 (31%) of patients with 6 (7%) patients reporting restrictions in the physical component summary, 16 (18%) in the mental component summary, and 6 (7%) in both components.

**Fig. 1:**
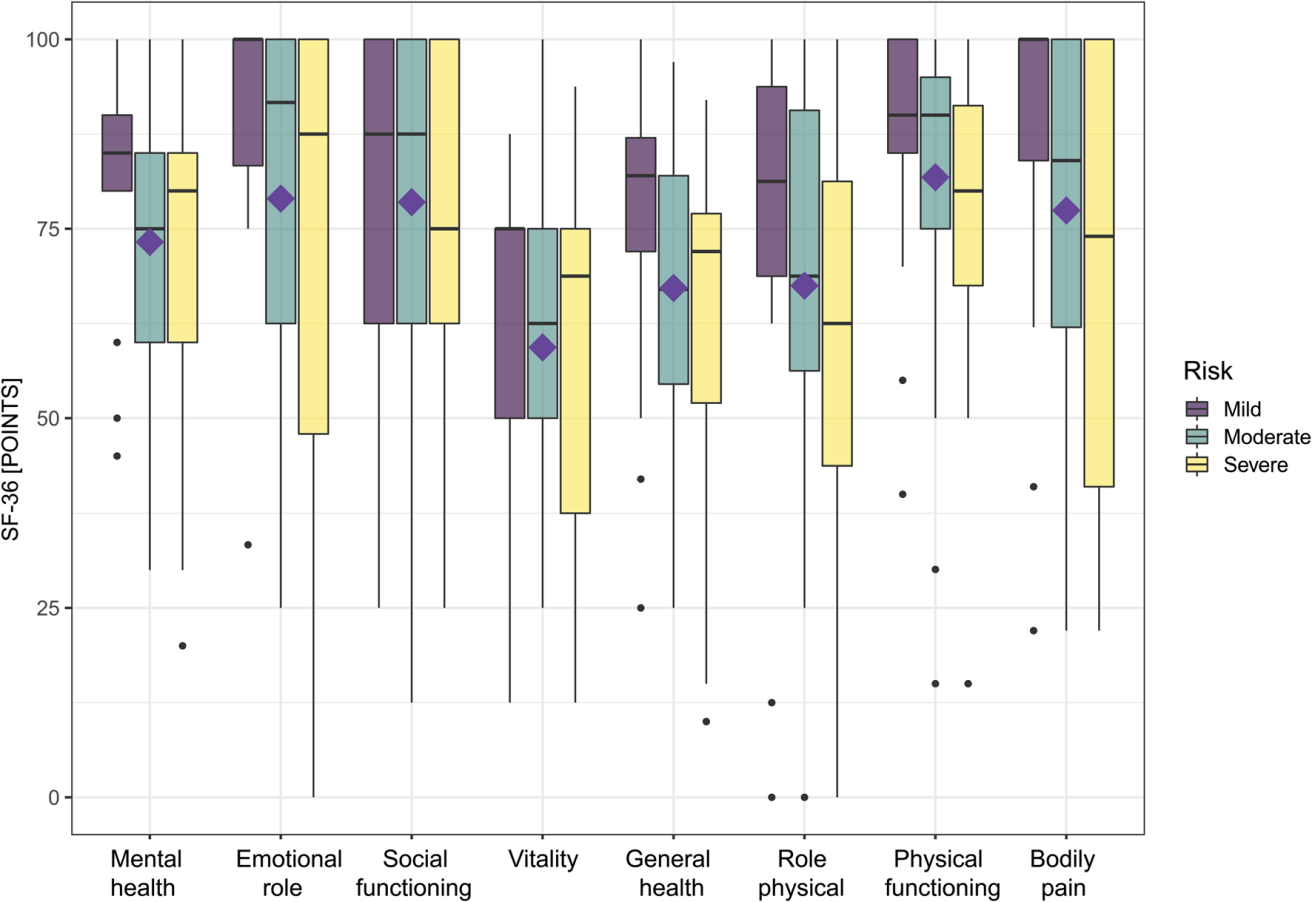
Box blots for each domain of the SF-36 stratified by disease severity during the acute phase of the disease. We did not find a significant difference across severity groups (*p* > 0.05). The central line shows the 50th percentile, the upper and lower lines the 75th and 25th percentile, and the lines end at the 90th and 10th percentiles. The rectangles indicate the median value of the whole cohort

Mean values of all domains of the SF-36 were within population normal ranges (> 40; Fig. 1). Still, 30/90 (33%) patients scored below 40 points in at least one of the following domains: physical functioning 3%, role physical 14%, bodily pain 11%, general health 9%, vitality 20%, social functioning 10%, role emotional 11%, and mental health 6%. The domains did not differ by initial disease severity (*p* > 0.05; Fig. 1).

### Mental health and SF-36 at the 3-month follow-up

Thirty percent of patients (*n* = 26/87) reported at least one mental health symptom at the 3-month follow-up. Twenty three % (*n* = 20) had signs of anxiety, 11% (*n* = 10) depression, and 10% (*n* = 9) reported PTSD. Co-occurrence of two mental health symptoms was evident in 3 (3%) patients, and 5 (6%) patients had all three. Eighteen (20%) patients were on antidepressants (*n* = 9, 10%), neuroleptics (*n* = 3, 3%), or sleep medication/benzodiazepines (*n* = 14, 16%) at the 3-month follow-up compared to 9 (10%) patients before COVID-19.

Patients with any mental health symptom had lower scores in the mental component summary compared to those without (*p* < 0.001); however, groups with and without mental health symptoms did not differ with regard to the physical component summary (*p* = 0.198).

**Fig. 2:**
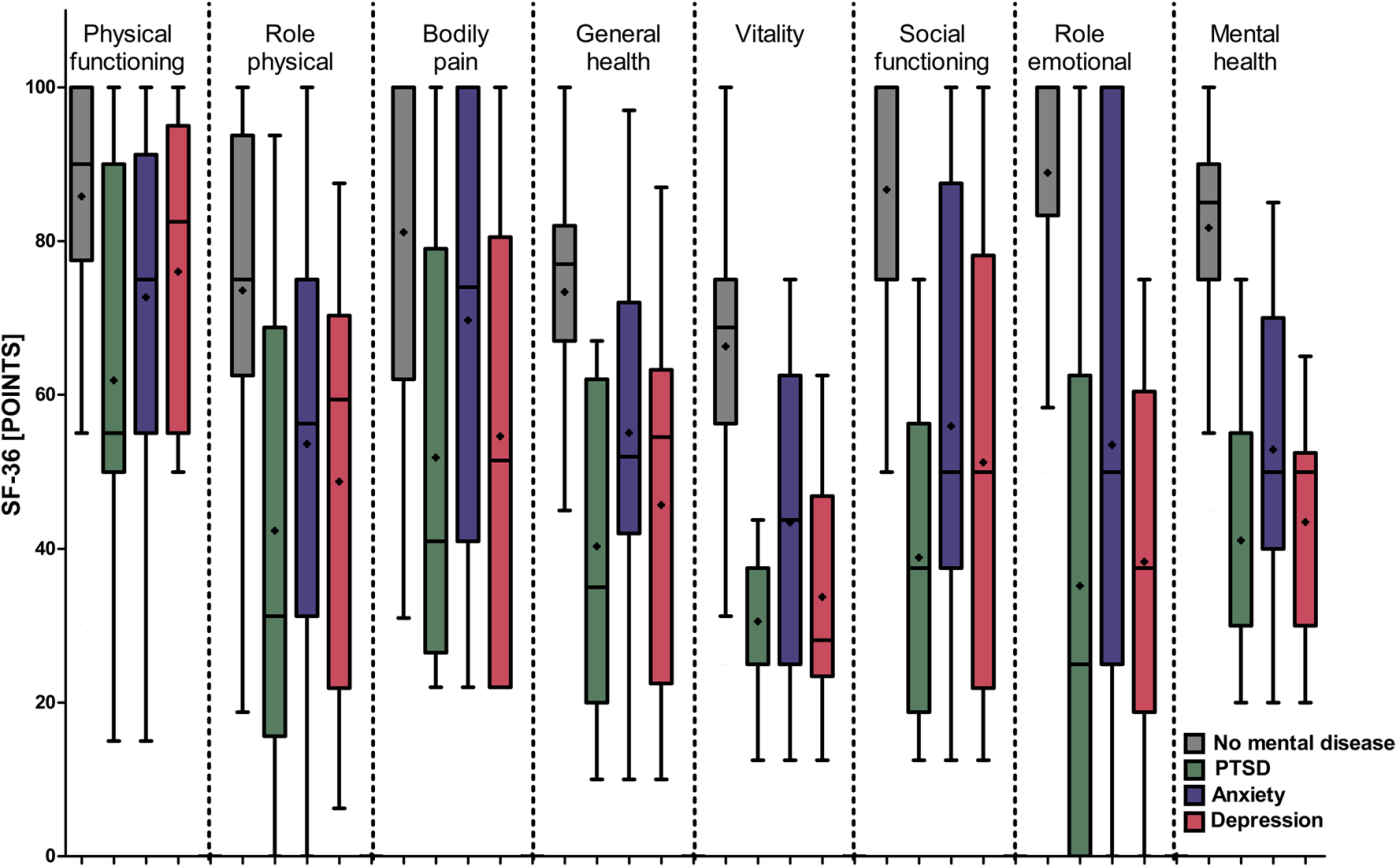
Box plots for each domain of the SF-36 stratified by mental health symptoms are displayed. Patients with mental health symptoms scored worse in all domains compared to patients without a mental health symptom (*p* < 0.05). The central line shows the 50th percentile, the upper and lower lines the 75th and 25th percentile, and the lines end at the 90th and 10th percentiles. The rectangles indicate the mean value

**Fig. 3:**
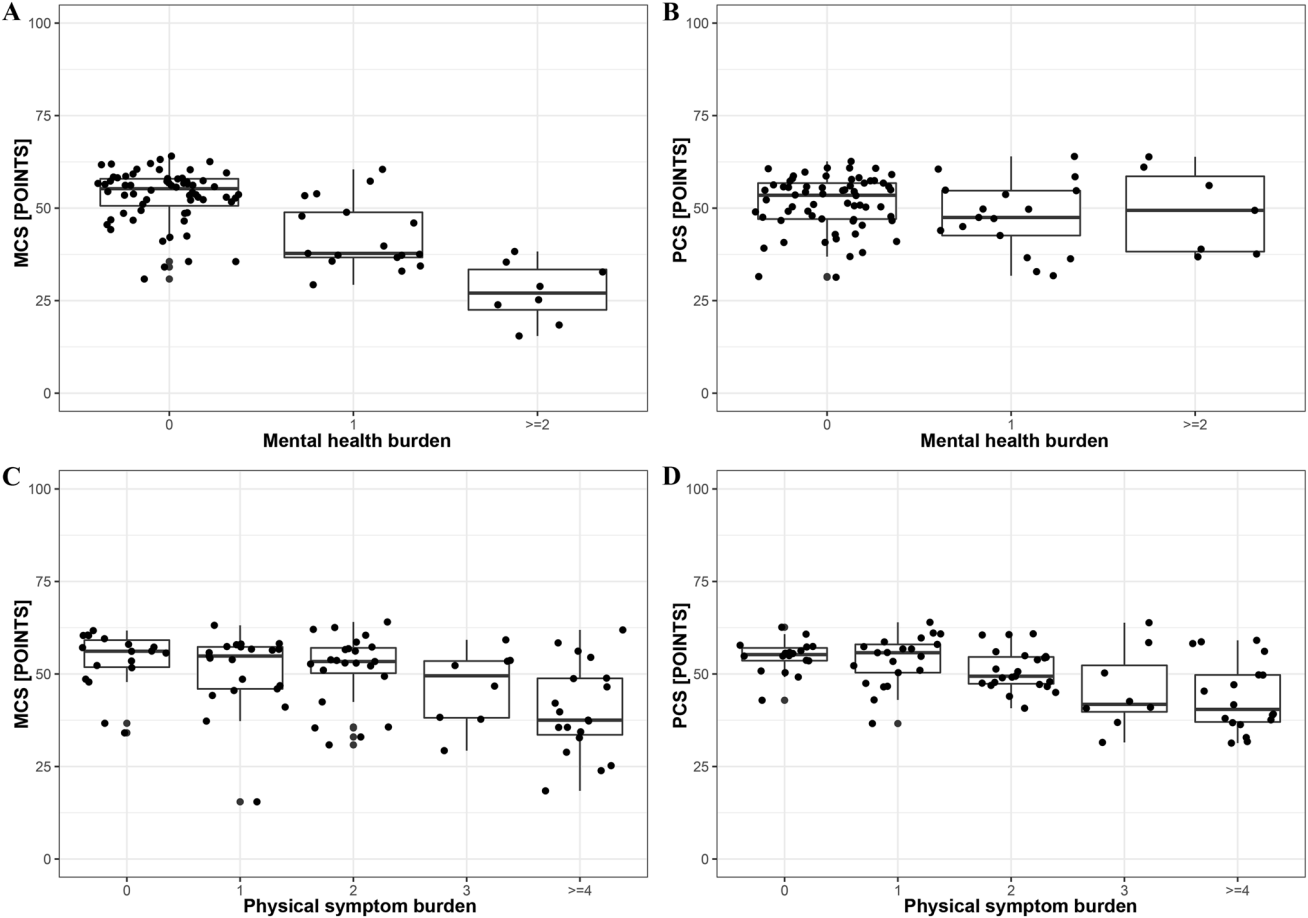
A higher mental health burden 3 months after COVID-19 was associated with a **A** lower mental component summary (MCS; *p* < 0.001) but not **B** with the physical component summary (PCS). A higher physical symptom burden 3 months after COVID-19 was associated with a lower **C** MCS (*p* < 0.001) and **D** PCS (*p* < 0.001)

Patients with any mental health symptom scored worse in all domains compared to patients without a mental health symptom (*p* < 0.05; Fig. 2). We found a cumulative dose effect linked to the number of mental health symptoms on the mental component summary: the more mental health symptoms the patients had, the lower was the MCS (IRR = 0.776, SE = 0.018, *p* < 0.001; Fig. 3A). However, the mental health burden was not associated with the PCS (*p* = 0.095; Fig. 3B).

### Physical symptom burden and SF-36 at the 3-month follow-up

The physical symptom burden at the 3-month follow-up ranged from 0 (*n* = 20, 22%) to 9 (*n* = 1, 1%) points (Supplemental Fig. 1). We found a negative association between the physical symptom burden and the physical component summary of the SF-36 (IRR = 0.958, SE = 0.008, *p* < 0.001) as well as the MCS (IRR = 0.957, SE = 0.008, *p* < 0.001; Fig. 3C, D).

### Factors associated with impaired quality of life at the 3-month follow-up

Factors associated with impaired HR-QoL, PCS < 40 and MCS < 40 in univariate analysis are given in Table 2.

**Table 2:** Univariate analysis of clinically meaningful parameters regarding impaired SF-36 < 40, MCS < 40, and PCS < 40

	SF-36 ≥ 40	SF-36 < 40	*p* value*	MCS ≥ 40	MCS < 40	*p* value*	PCS ≥ 40	PCS < 40	*p* value*
*n*	61	28		67	22		76	12	
**Demographics**									
Age	57.1 (12.6)	53.5 (11.9)	0.22	57.3 (12.4)	52.0 (11.8)	0.08	56.13 (12.4)	55.00 (13.8)	0.77
Body mass index	26.7 (5.2)	26.6 (4.7)	0.95	26.4 (5.1)	27.4 (4.6)	0.40	27.1 (5.0)	24.35 (4.0)	0.08
Male sex	36 (59.0)	19 (67.9)	0.57	39 (58.2)	16 (72.7)	0.34	49 (64.5)	6 (50.0)	0.52
Current smoker	0 (0.0)	2 (7.1)	0.18	1 (1.5)	1 (4.5)	0.99	0 (0.0)	2 (16.7)	0.01
**Premedical history**									
Cardiovascular disease	21 (34.4)	11 (39.3)	0.84	24 (35.8)	8 (36.4)	1.00	27 (35.5)	5 (41.7)	0.93
Pulmonary disease	11 (18.0)	6 (21.4)	0.93	11 (16.4)	6 (27.3)	0.42	13 (17.1)	3 (25.0)	0.80
Diabetes mellitus II	8 (13.1)	6 (21.4)	0.49	9 (13.4)	5 (22.7)	0.48	12 (15.8)	2 (16.7)	1.00
Malignancy	9 (14.8)	3 (10.7)	0.85	10 (14.9)	2 (9.1)	0.74	11 (14.5)	1 (8.3)	0.90
Neurological disease	14 (22.6)	7 (25.0)	0.79	15 (22.1)	6 (27.3)	0.77	17 (22.1)	4 (33.3)	0.45
**Treatment and hospital course**									
Risk category			0.25			0.51			0.24
Mild (Outpatient)	14 (23.0)	3 (10.7)		14 (20.9)	3 (13.6)		15 (19.7)	2 (16.7)	
Moderate (Hospitalized)	35 (57.4)	16 (57.1)		39 (58.2)	12 (54.5)		46 (60.5)	5 (41.7)	
Severe (ICU)	12 (19.7)	9 (32.1)		14 (20.9)	7 (31.8)		15 (19.7)	5 (41.7)	
Risk category (WHO)			0.35			0.28			0.18
Mild	14 (23.0)	3 (10.7)		14 (20.9)	3 (13.6)		15 (19.7)	2 (16.7)	
Moderate	17 (27.9)	6 (21.4)		20 (29.9)	3 (13.6)		19 (25.0)	4 (33.3)	
Severe	18 (29.5)	10 (35.7)		19 (28.4)	9 (40.9)		27 (35.5)	1 (8.3)	
Critical	12 (19.7)	9 (32.1)		14 (20.9)	7 (31.8)		15 (19.7)	5 (41.7)	
Mechanical ventilation	11 (18.0)	9 (32.1)	0.23	13 (19.4)	7 (31.8)	0.36	14 (18.4)	5 (41.7)	0.15
Steroid use	7 (11.5)	8 (28.6)	0.09	9 (13.4)	6 (27.3)	0.24	11 (14.5)	4 (33.3)	0.23
Length of hospital stay (days)**	9.8 (10.4)	16.5 (17.5)	0.03	10.9 (12.1)	14.8 (16.4)	0.24	10.0 (9.9)	23.7 (23.7)	0.00
ICU days	3.2 (7.4)	6.2 (11.9)	0.14	3.6 (7.8)	5.9 (12.2)	0.29	2.8 (6.9)	11.8 (16.1)	0.00
**Mental health and cognition at 3 months**									
HADS-A	3.1 (2.4)	7.64 (4.1)	0.00	3.3 (2.6)	8.4 (4.0)	0.00	4.0 (3.1)	7.8 (5.4)	0.00
HADS-A > 7	4 (6.9)	15 (53.6)	0.00	5 (7.8)	14 (63.6)	0.00	13 (17.8)	5 (41.7)	0.14
HADS-D	1.7 (1.7)	5.4 (3.9)	0.00	1.8 (1.7)	6.3 (3.9)	0.00	2.5 (2.6)	5.3 (5.0)	0.01
HADS-D > 7	1 (1.7)	9 (32.1)	0.00	1 (1.6)	9 (40.9)	0.00	6 (8.2)	4 (33.3)	0.04
PCL-5 > 32	1 (1.7)	8 (28.6)	0.00	1 (1.6)	8 (36.4)	0.00	4 (5.5)	4 (33.3)	0.01
Any mental health disorder	6 (10.3)	19 (67.9)	0.00	7 (10.9)	18 (81.8)	0.00	17 (23.3)	7 (58.3)	0.03
Fatigue	10 (16.4)	12 (44.4)	0.01	13 (19.7)	9 (40.9)	0.09	12 (15.8)	9 (81.8)	0.00
Sleep disturbance	11 (18.0)	19 (67.9)	0.00	15 (22.4)	15 (68.2)	0.00	19 (25.0)	10 (83.3)	0.00
Impaired concentration	7 (11.5)	11 (42.3)	0.00	8 (12.1)	10 (47.6)	0.00	14 (18.7)	3 (27.3)	0.79
MOCA	27.8 (2.0)	26.9 (2.6)	0.11	27.9 (2.0)	26.5 (2.6)	0.01	27.4 (2.3)	28.1 (1.5)	0.36
MOCA < 26	9 (15.5)	7 (26.9)	0.35	9 (14.5)	7 (31.8)	0.14	15 (20.5)	1 (10.0)	0.72
**Neurological signs and diseases at 3 months**									
Any new neurological disease	7 (11.5)	4 (14.3)	0.98	9 (13.4)	2 (9.1)	0.87	7 (9.2)	4 (33.3)	0.06
New peripheral neuromyopathy	6 (9.8)	3 (10.7)	1.00	7 (10.4)	2 (9.1)	1.00	6 (7.9)	3 (25.0)	0.19
CIP/CIM	0 (0.0)	3 (10.7)	0.05	1 (1.5)	2 (9.1)	0.30	0 (0.0)	3 (25.0)	0.00
Any neurological sign	33 (54.1)	17 (60.7)	0.72	38 (56.7)	12 (54.5)	1.00	41 (53.9)	9 (75.0)	0.29
Objective hyposmia (SS-16 ≤ 12)	21 (36.2)	14 (51.9)	0.26	25 (39.7)	10 (45.5)	0.82	27 (37.0)	8 (72.7)	0.06
Subjective hyposmia	11 (18.0)	3 (10.7)	0.57	13 (19.4)	1 (4.5)	0.19	12 (15.8)	2 (16.7)	1.00
Subjective hypogeusia	6 (9.8)	4 (14.3)	0.80	8 (11.9)	2 (9.1)	1.00	7 (9.2)	3 (25.0)	0.27
New headache	2 (3.3)	2 (7.1)	0.79	2 (3.0)	2 (9.1)	0.54	2 (2.6)	1 (8.3)	0.88
Vertigo	2 (3.3)	4 (14.3)	0.14	3 (4.5)	3 (13.6)	0.32	2 (2.6)	3 (25.0)	0.02
Self-reported numbness	8 (13.8)	11 (44.0)	0.01	10 (15.6)	9 (47.4)	0.01	14 (19.4)	4 (40.0)	0.29
Gait abnormality	1 (1.6)	3 (10.7)	0.17	3 (4.5)	1 (4.5)	1.00	2 (2.6)	2 (16.7)	0.16
Myalgia	4 (6.6)	4 (14.3)	0.43	7 (10.4)	1 (4.5)	0.68	4 (5.3)	4 (33.3)	0.01
Tremors	5 (8.2)	2 (7.1)	1.00	5 (7.5)	2 (9.1)	1.00	7 (9.2)	0 (0.0)	0.60
Muscle atrophy	1 (1.6)	2 (7.1)	0.48	2 (3.0)	1 (4.5)	1.00	1 (1.3)	2 (16.7)	0.06
Paresis	2 (3.3)	3 (10.7)	0.36	4 (6.0)	1 (4.5)	1.00	3 (3.9)	2 (16.7)	0.27
**Cardio-pulmonary health at 3 months**									
CT pathology	36 (61.0)	19 (70.4)	0.55	40 (62.5)	15 (68.2)	0.83	47 (63.5)	7 (63.6)	1.00
CT abnormalities > 5	15 (25.0)	9 (33.3)	0.59	18 (27.7)	6 (27.3)	1.00	18 (24.0)	6 (54.5)	0.08
CT abnormalities > 10	5 (8.3)	5 (18.5)	0.31	5 (7.7)	5 (22.7)	0.13	7 (9.3)	3 (27.3)	0.22
Reduced DLCO	9 (15.0)	5 (18.5)	0.92	12 (18.2)	2 (9.5)	0.55	10 (13.3)	4 (36.4)	0.14
Impaired lung function	21 (35.0)	11 (39.3)	0.88	25 (37.9)	7 (31.8)	0.80	23 (30.7)	8 (66.7)	0.04
Diastolic dysfunction	34 (56.7)	16 (59.3)	1.00	37 (56.9)	13 (59.1)	1.00	43 (57.3)	6 (54.5)	1.00
Persistent dyspnoa	18 (30.0)	13 (48.1)	0.16	22 (33.8)	9 (40.9)	0.73	23 (30.7)	8 (72.7)	0.02
Dyspnoea, mMRC			0.19			0.39			0.00
0	42 (70.0)	13 (50.0)		43 (66.2)	12 (57.1)		52 (69.3)	2 (20.0)	
1	16 (26.7)	9 (34.6)		19 (29.2)	6 (28.6)		21 (28.0)	4 (40.0)	
2	1 (1.7)	2 (7.7)		2 (3.1)	1 (4.8)		1 (1.3)	2 (20.0)	
3	1 (1.7)	1 (3.8)		1 (1.5)	1 (4.8)		1 (1.3)	1 (10.0)	
4	0 (0.0)	1 (3.8)		0 (0.0)	1 (4.8)		0 (0.0)	1 (10.0)	
Persistent cough	8 (13.3)	6 (22.2)	0.47	9 (13.8)	5 (22.7)	0.52	10 (13.3)	4 (36.4)	0.14
**Laboratory parameters at 3 months**									
Hyperferrtinemia	10 (16.7)	6 (22.2)	0.75	12 (18.5)	4 (18.2)	1.00	13 (17.3)	3 (27.3)	0.71
HbA1c			0.47			0.54			0.99
< 5.7%	40 (66.7)	17 (63.0)		42 (64.6)	15 (68.2)		49 (65.3)	7 (63.6)	
≥ 5.7% and < 6.5%	16 (26.7)	6 (22.2)		18 (27.7)	4 (18.2)		19 (25.3)	3 (27.3)	
≥ 6.5%	4 (6.7)	4 (14.8)		5 (7.7)	3 (13.6)		7 (9.3)	1 (9.1)	
NT elevated	15 (25.9)	6 (22.2)	0.93	16 (25.4)	5 (22.7)	1.00	17 (23.3)	4 (36.4)	0.58
D dimer elevated	14 (23.7)	5 (18.5)	0.79	17 (26.6)	2 (9.1)	0.16	15 (20.3)	4 (36.4)	0.42
CRP levels elevated	5 (8.3)	3 (11.1)	0.99	5 (7.7)	3 (13.6)	0.68	7 (9.3)	1 (9.1)	1.00
IL6 elevated	4 (6.7)	1 (3.7)	0.96	5 (7.7)	0 (0.0)	0.42	4 (5.3)	1 (9.1)	1.00
**Functional outcome at 3 months**									
GOSE			0.07			0.37			0.00
5	0 (0.0)	1 (3.6)		1 (1.5)	0 (0.0)		0 (0.0)	1 (8.3)	
6	2 (3.3)	3 (10.7)		3 (4.5)	2 (9.1)		2 (2.6)	3 (25.0)	
7	18 (29.5)	12 (42.9)		20 (29.9)	10 (45.5)		23 (30.3)	6 (50.0)	
8	41 (67.2)	12 (42.9)		43 (64.2)	10 (45.5)		51 (67.1)	2 (16.7)	
mRS			0.01			0.14			0.00
0	37 (60.7)	10 (35.7)		39 (58.2)	8 (36.4)		45 (59.2)	2 (16.7)	
1	21 (34.4)	10 (35.7)		22 (32.8)	9 (40.9)		27 (35.5)	3 (25.0)	
2	3 (4.9)	7 (25.0)		5 (7.5)	5 (22.7)		4 (5.3)	6 (50.0)	
4	0 (0.0)	1 (3.6)		1 (1.5)	0 (0.0)		0 (0.0)	1 (8.3)	

Data are given in *n* (%) or mean (SD)

*χ^2^, *t* test or, Mann–Whitney *U* test were used to assess for differences across the indicated groups. A *p* value < 0.05 signifies significantly different data distribution across the groups

**In non-hospitalized patients, “0” was used for calculation

*SS-16* 16-item Sniffin' Sticks test, *DLCO* diffusion capacity for carbon monoxide, *mMRC* Modified British Medical Research Council, *GOSE* Glasgow Outcome Scale Extended, *mRS* modified Rankin Scale Score, *HADS* Hospital Anxiety and Depression Scale, *PCL-5* Post-traumatic Stress Disorder Checklist-5

**Fig. 4:**
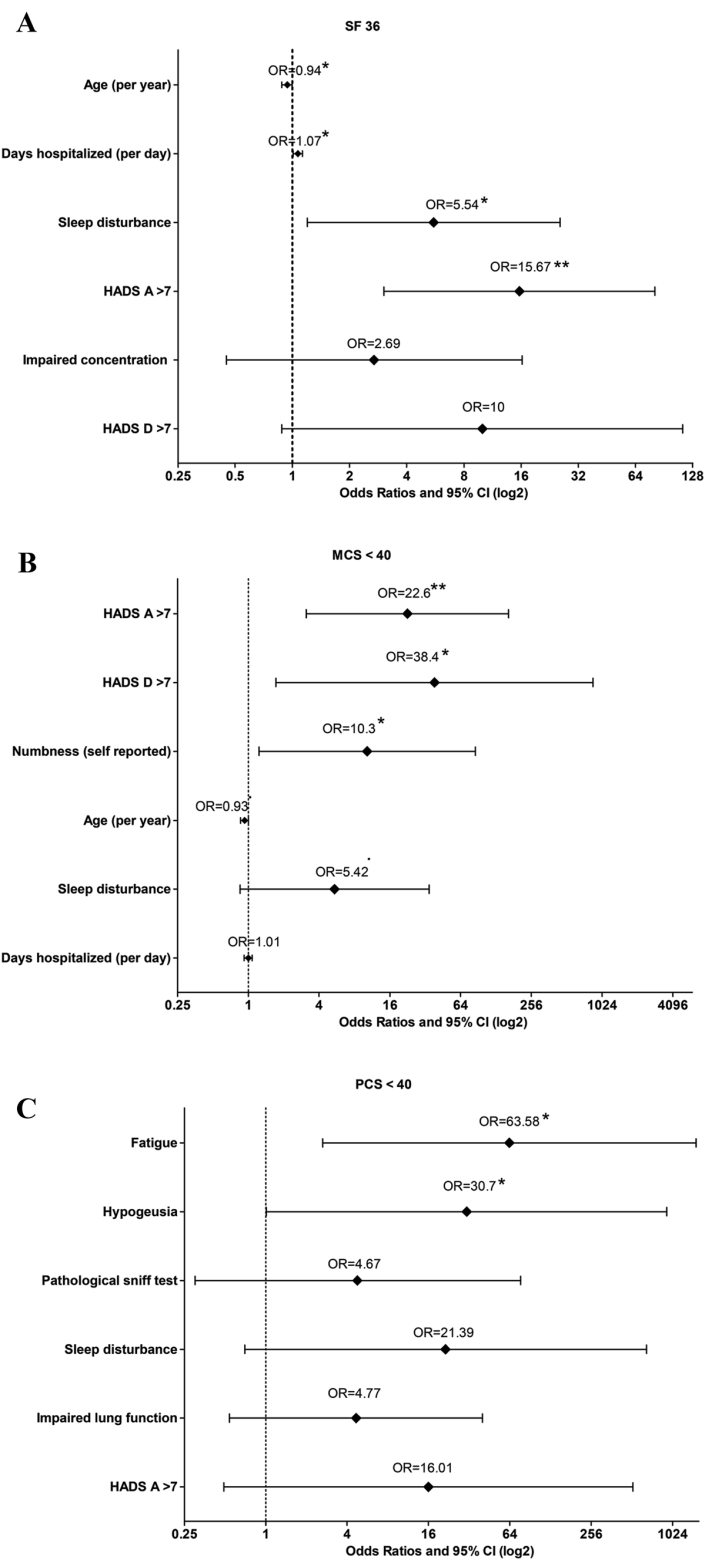
Factors associated with impaired **A** SF-36 < 40, **B** mental component summary (MCS) < 40, and **C** physical component summary (PCS) < 40 with calculated adjusted odds ratios based on the logistic regression with the 95% confidence intervals are shown. HADS-A > 7 is indicative of the presence of anxiety, HADS-D > 7 of depression. In non-hospitalized patients, “0” days were used for calculation

Factors associated with impaired overall HR-QoL in multivariable analysis included the presence of sleep disturbance [adjOR (95%-CI) 5.54 (1.2–25.61), *p* = 0.028], the presence of anxiety [adjOR (95%CI) 15.67 (3.03–80.99), *p* = 0.001], younger age [per year, adjOR (95%CI) 0.94 (0.88–1), *p* = 0.0499], and prolonged days of hospitalization during the acute disease course per day, adjOR (95%CI) 1.07 (1.01–1.13), *p* = 0.015; Fig. 4A].

The presence of anxiety [adjOR (95%CI) 22.59 (1.11–163.78), *p* = 0.002] or depression [adjOR (95%CI) 38.45 (1.72–858.18), *p* = 0.021], and self-reported numbness [adjOR (95%CI) 10.27 (1.23–85.67), *p* = 0.031] were associated with MCS < 40 in multivariable analysis (Fig. 4B).

Factors associated with PCS < 40 in multivariable analysis included fatigue [adjOR (95%CI) 3.58 (2.64–1529.56), *p* = 0.011], and hypogeusia [adjOR (95%CI) 30.7 (1.01–929.06), *p* = 0.049) at 3 months (Fig. 4C).

## Discussion

In this multicentre prospective cohort study, we found that younger age, prolonged hospitalization due to COVID-19 as well as current sleep disturbance, anxiety, and depression contributed to impaired health-related QoL in hospitalized and non-hospitalized COVID-19 survivors 3 months after primary diagnosis. Patients reporting any mental health symptom scored worse in all domains of the SF-36 relative to those without, suggesting a predominant impact of mental health on long-term quality of life after COVID-19.

Every third patient reported impaired QoL 3 months after COVID-19, which is in the lower range compared to previous reports (33% to 72% at 3 months) [12, 14]. This difference may be explained by the variety of tests used to assess QoL and selective patient populations studied. We included consecutive COVID-19 patients across the whole severity spectrum, whereas many other studies focused on hospitalized patients only [5, 10, 30]. In contrast to previous reports [5, 31], restrictions in HR-QoL were not associated with disease severity, which underlines the socioeconomic impact of COVID-19 even in mildly affected patients who do not require hospital admission. Still, one would expect an association between persistent dyspnoea or impaired lung function and an impaired physical health component of the SF-36 in COVID-19 patients who required mechanical ventilation [31]. We only found that a prolonged hospital stay, indicative of a more severe disease course, was associated with impaired quality of life. In this regard, it is important to mention that the majority of severe grade patients in our cohort underwent early and multimodal rehabilitation which had a positive effect on the physical performance, lung function, and daily activities as we could show previously [32]. Therefore, multi-disciplinary rehabilitation may have contributed to a better physical health status in this patient group [32]. Furthermore, the results of the CoviLD study indicated a significant improvement of physical symptoms and pulmonary damages over the first 3 months after COVID-19 diagnosis in our patients [22]. In line with our findings, a study conducted in severe acute respiratory syndrome (SARS) survivors did not demonstrate a difference in the domains of QoL among ICU and non-ICU patients [33].

Strikingly, impairments were higher for the mental than for the physical well-being in our study which is in accordance with another study in COVID-19 patients [14]. Similarly, a study demonstrated significant mental health deficits and restrictions in the mental component of the SF-36 in survivors of the epidemic Influenza A (H7N9) [18] although psychiatric disorders, dementia, and insomnia may be even more prevalent in COVID-19 survivors compared to patients after influenza, or other respiratory tract infections [8].

Restrictions in the individual’s life during the pandemic may also have contributed to the relatively high prevalence of impaired HR-QoL independent of the disease. This seems to apply especially to mental well-being including anxiety, depression, and PTSD [34–38]. In a questionnaire-based study of almost 15 000 participants recruited in 14 countries including Austria, the authors found a decline of the overall mental well-being in 73% of respondents during the pandemic. Signs of depression rose from 14% in the pre-COVID era to 45% during the pandemic [34]. In another study of Austrian respondents, 21% reported depressive symptoms, and 19% had signs of anxiety during the pandemic [35]. Although prevalence rates are highly comparable to our post-COVID population, where symptoms of anxiety, depression, and PTSD were reported in 25%, 11%, and 11%, respectively, questionnaire-based online surveys are prone to selection bias and may, therefore, overestimate the true prevalence. Contrastingly, our data are equally comparable to other reports with similar prevalence rates of depression, anxiety, and PTSD after COVID-19 [10]. The only way to distinguish whether COVID-19 is responsible for these symptoms is to compare patients after COVID-19 with those without.

Importantly, the cumulative mental health burden expressed by co-occurrence of anxiety, depression, and PTSD largely determined the mental component of the SF-36 in our study. This underlines the necessity to screen post-COVID-19 patients for anxiety, depression, and PTSD to early identify those who require targeted treatments in order to influence quality of life in the long term.

In comparison, a higher physical symptom burden 3 months after COVID-19 was correlated with an impaired score on the physical and mental components of the SF-36. Some of these symptoms, such as poor concentration, impaired lung function, and polyneuromyopathy may potentially be modifiable and are susceptible to supportive interdisciplinary concepts of therapy and early rehabilitation measures. However, the natural history of these symptoms is incompletely studied, and specific management strategies are not available. Not surprisingly, these long-lasting symptoms also have an impact on the subjective quality of life, even though the results of our study suggest that mental health symptoms may be even more important for HR-QoL.

Sleep disturbance was also associated with impaired QoL after COVID-19. Sleep disturbance is a frequent symptom after COVID-19 [5] and an abnormal muscle activity during REM sleep may even reflect a potential underlying CNS pathology secondary to the Sars-CoV-2 infection [39]. However, sleep quality may be decreased secondary to pandemic-related confinements, irrespective of acute COVID-19, with higher levels of stress experienced during the pandemic, i.e. when people fear to lose their work [35].

Interestingly, younger patients more often reported impaired HR-QoL. A possible explanation may be the higher impact of the pandemic on younger individuals [34]. Several studies suggest that particularly young females with low incomes and unemployed people are susceptible for mental health issues due to the pandemic [35, 40–42].

The only factors independently associated with lower scores on the physical domain of the SF-36 included hypogeusia and fatigue. Persistent fatigue with exhaustion and malaise is a common symptom after COVID-19 and was reported by 50% of patients 2 months after the disease [11] and still 14% complained about fatigue 6 months after COVID-19 in a mixed patient population encompassing mild to severe patients [12]. Persistent fatigue after COVID-19 may be triggered by low-level inflammation secondary to the infection [43], however underlying mechanisms are not yet fully understood, and results are not convincing [44]. Therefore, further studies are needed to develop effective treatment strategies to attenuate fatigue syndromes in order to positively influence the subjective well-being. Our observation corresponds to findings in patients after ARDS, where persisting weakness and neuropsychological impairments rather than impaired lung function per se were associated with impaired QoL [45].

Some limitations to the study merit consideration. First, we lack a control group without COVID-19, and we, therefore, do not know whether diminished HR-QoL was secondary to COVID-19 or related to public health measurements during the pandemic. We, therefore, cannot establish a causal link. Second, we did not assess HR-QoL prior to or during COVID-19 and cannot conclude on the natural history of QoL. Third, despite consistent assessment of all patients 3 months after diagnosis, recovery times differed among patients and may have influenced HR-QoL. Still, we corrected for disease severity during the acute phase of disease. Fourth, the number of patients included in our study may have been too small to identify all relevant associated factors and the ones that were identified leave a degree of uncertainty of the precise relative risk reflected in high confidence intervals. To confirm our findings, a larger cohort is needed.

## Conclusions

In summary, our data suggest that HR-QoL is impaired in one third of post-COVID-19 patients across all severity groups. Mental health symptoms including anxiety, depression, and post-traumatic disorders largely determine quality of life and need special attention in the care after COVID-19.

## Supplementary Information

Below is the link to the electronic supplementary material.

Supplementary file1 (TIF 1188 kb)

## Data Availability

The data that support the findings of this study are available from the corresponding authors, upon reasonable request.
