# Supplementary figures and images for: Factors associated with impaired quality of life three months after being diagnosed with COVID-19
Source: Qual Life Res. 2021 Sep 28;31(5):1401–14. doi: 10.1007/s11136-021-02998-9 (PMC8476326; doi:10.1007/s11136-021-02998-9)

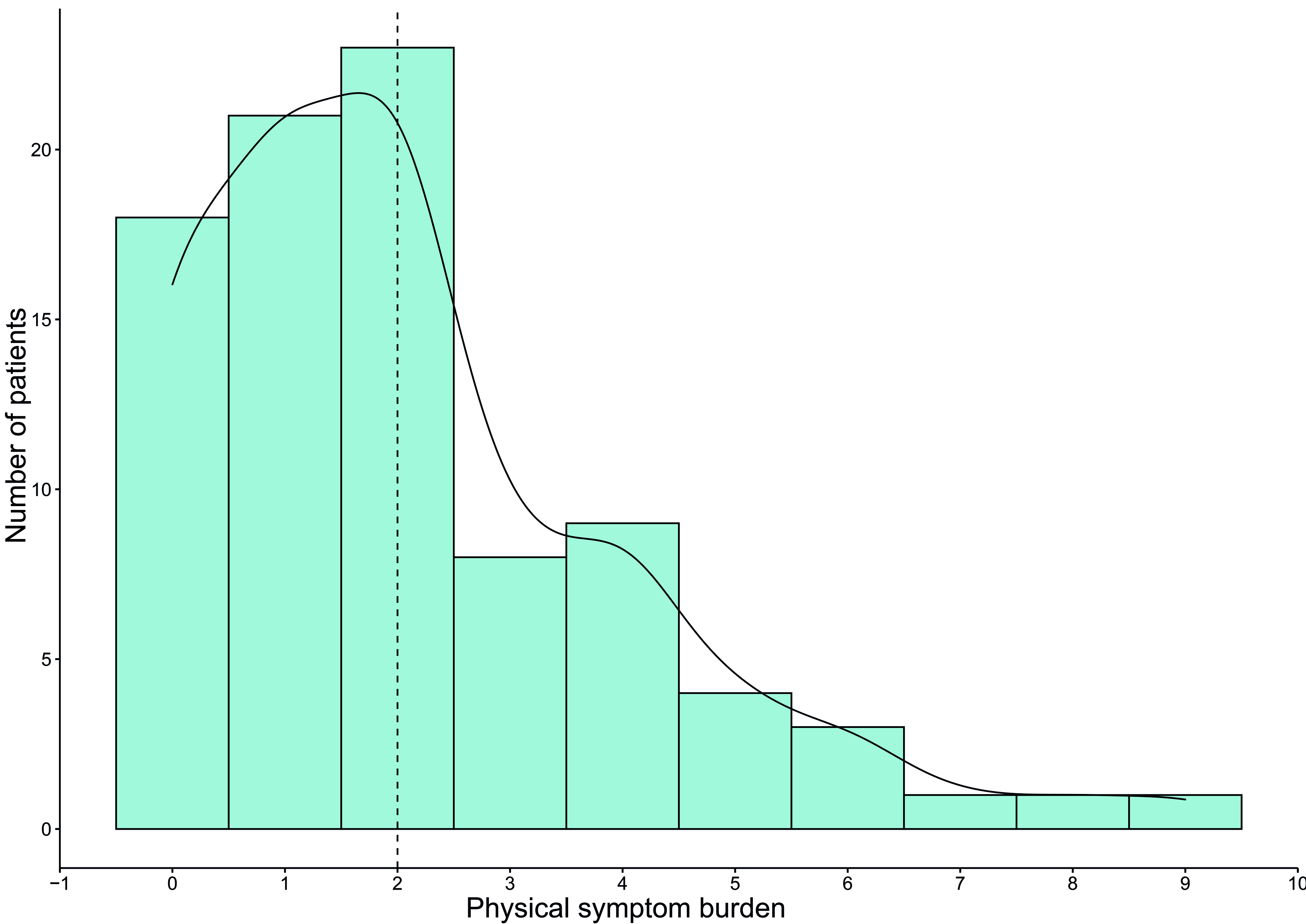

Supplement: Supplementary file 1 — Supplementary file1 (TIF 1188 kb) [file 11136_2021_2998_MOESM1_ESM.tif]
